# Determinants of breast self-examination practice among women attending pastoralist health facilities, Southern Ethiopia: a cross-sectional study

**DOI:** 10.1186/s12905-023-02158-w

**Published:** 2023-01-11

**Authors:** Eskinder Israel, Nefsu Awoke, Tagese Yakob, Amdehiwot Aynalem, Alemayehu Talto, Kibrework Bezabih

**Affiliations:** 1 School of Midwifery, College of Health Science and Medicine, Wolaita Sodo University, Wolaita Sodo, Ethiopia; 2 School of Nursing, College of Health Science and Medicine, Wolaita Sodo University, Wolaita Sodo, Ethiopia; 3 Bele Hawassa Primary Hospital, Bele Hawassa Town Administration, Wolaita, Ethiopia; 4 School of Nursing, College of Medicine and Health Science, Hawassa University, Hawassa, Ethiopia

**Keywords:** Breast self-examination, Breast self-examination practices, Pastoralists, Southern Ethiopia

## Abstract

**Background:**

Breast cancer remains the most serious public health problem affecting millions of women worldwide. Despite various studies regarding breast self-examination practices conducted among health professionals and students in Ethiopia, limited information was found on women attending health care services in the pastoralist community. This study aimed to identify the determinants of breast self-examination practice (BSE) among women attending pastoralist health facilities in Southern Ethiopia.

**Methods:**

An institutional-based cross-sectional study was conducted among 421 women who were attending family planning services in pastoralist health facilities in South Omo Zone, Southern Ethiopia from January to February 2022 using systematic random sampling to select a woman from each health facility in Jinka town. Data were collected using structured, pretested, and interviewer-administered questionnaires. Descriptive statistics such as proportions, means, and standard deviations were used to describe each relevant variable. Bivariate and multivariate logistic regression analyses were used to identify the determinants of BSE practices among women.

**Result:**

The mean age of the women was 25.43 ± 6.66 years. Fifty-four percent (n = 173) of the women had heard of BSE from health professionals. Eighty-nine (21.1%) women had undergone BSE. Women who resided in the urban areas (AOR = 6.79, CI: 3.40, 13.56), attained at least primary education and above (AOR = 8.96, CI: 4.14, 19.35), heard about BSE (AOR = 4.07, CI: 2.07, 7.98), and had a family history of breast cancer (AOR = 7.46, CI = 3.27, 17.00) were significantly associated with BSE practice.

**Conclusion and recommendation:**

Our study showed that women's practice of BSE was lower when compared with the local studies. We recommend health care professionals and others working in the area improve ways of increasing awareness about breast cancer, including its risk, and the need for BSE.

## Introduction

In the lens of Sustainable Development Goals (SDGs), Goal three aims to prevent non-communicable diseases (NCDs) by nearly one-third [1]. Worldwide, more than 2.3 million women of reproductive age are diagnosed with breast carcinoma, and nearly 685,000 deaths from breast cancer have been reported in 2020 [2]. Despite its varied prevalence, breast cancer remains the most serious public health problem affecting millions of women in the world [3]. Sub-Saharan African (SSA) countries, where there is limited access to healthcare suffer a disproportionate burden of this pandemic yielding an age-adjusted rate of 28/100, 000 women [4].

To prevent further damage, the American cancer society (ACS) designed and recommended three main screening methods to decrease the prevalence of breast cancer morbidity and mortality such as mammography, clinical breast examination (CBE), and Breast self-examination (BSE). Among these screening methods, BSE is ideal for use in resource-limited settings including Ethiopia due to its easy access, low cost, and simplicity when compared with other screening approaches [5]. Despite being the leading public health problem in Ethiopia, the Federal Ministry of Health (FMOH) has been aggressively working to combat communicable and non-communicable diseases including breast cancer over the past few decades [6].

Varying levels of practice have been reported among different population groups and within the regions in Ethiopia. According to a recent study conducted in 2021, the Gambella region reported the highest prevalence of BSE (61.5%) whereas the lowest was in the Tigray region (21.2%) [7]. Cross-sectional studies conducted among female students at Gondar, Debre Berhan University, and west Arsi indicated a prevalence of 17.4%, 28.3%, and 31.5% prevalence of BSE practice respectively [8–10]. Another study from the Debre tabor, western shoa, Gamo Gofa, and western Ethiopia among employed women showed 28.3%, 32.6%, 34.3%, and 77% of BSE prevalence respectively [11–14].

The practice of BSE among women who attend care in health facilities also varies. Prevalence rates of 6.5%, 20.5%, 28.4%, and 58.6% were reported in the public health facilities of Adwa, Modjo, Nigeria, and Uganda respectively [15–18]. These disparities in the level of BSE practices among women call for urgent action.

Due to the lack of effective early screening methods in resource-limited settings including Ethiopia, most breast cancer cases will progress to an advanced stage. This makes treatment modalities ineffective for women due to various reasons [19, 20]. Early detection and treatment of breast cancer are crucial for better treatment outcomes [1, 5]. If not, it results in decreased quality of life and increases the possibility of developing complications [1, 2]. It is also known that women in pastoralist communities have their own special and specific contexts such as social norms, culture, beliefs, attitudes, and values that can affect them from not seeking health and their overall health status.

Although various studies have been conducted on BSE practices among women, health professionals, and students in Ethiopia, limited information was found on women attending health care services in the pastoralist community. Most studies conducted have solely focused on urban settings, referral and tertiary hospitals, and school setups (including universities) that have access to information, education, and health care. However, women who were socially marginalized including the pastoralist community were neglected. Therefore, this study primarily aims to contribute to the knowledge base by assessing the women's practice of BSE in pastoralist health facilities in Southern Ethiopia, in 2022.

## Methods

### Study design, period, and setting

An institutional-based cross-sectional study was conducted among women who were attending family planning (FP) services in Pastoralist health facilities in South Omo Zone, Southern Ethiopia from January to February 2022. This study was conducted in the pastoralist health facilities of South Omo, the zonal structure of Southern Ethiopia. The South Omo Zone is principally characterized by socially marginalized communities with the lowest socioeconomic status as well as dominated by nomadic and pastoralist ways of life. It has a diverse ethnic group that contains around 21 different tribes. It is also one of the border zones, and 750 km from the center of Addis Ababa, the capital of Ethiopia. Jinka town, the Capital of South Omo has 3 governmental health facilities such as; Jinka General Hospital (JGH), Millennium Health center, and Bethemal Health center.

### Population

All women who visited governmental health facilities and were aged 20 years and above were the source population. All systematically selected women aged 20 years and above were the study population. Women who were 20 years and above, and had no history of breast-related diseases were included in the study. Women who were ill and didn't want to participate, and were not mentally sound were excluded.

#### Sample size determination

The sample size was calculated using the single population proportion formula. Different assumptions were used to calculate the sample size such as; a 95% confidence level, 5% margin of error, and 53.6% proportion of women who use BSE in Northern Ethiopia [21].

$${\text{n}} = \frac{{{\text{Z}}^{2} {\text{P}}\left( {1 - {\text{P}}} \right)}}{{{\text{W}}^{2} }}$$

where:—n = required sample size, Z (confidence interval of 95% which is = 1.96), P—the estimated proportion, W—Marginal error

$$\begin{aligned} & {\text{n}}_{{1}} = \frac{{\left( {1.96} \right)^{2} { }\left( {0.5} \right)\left( {1 - 0.5{ }} \right)}}{{\left( {0.05} \right)^{2} }} \\ & {\text{n}}_{{1}} = { 384} \\ \end{aligned}$$

**Fig. 1:**
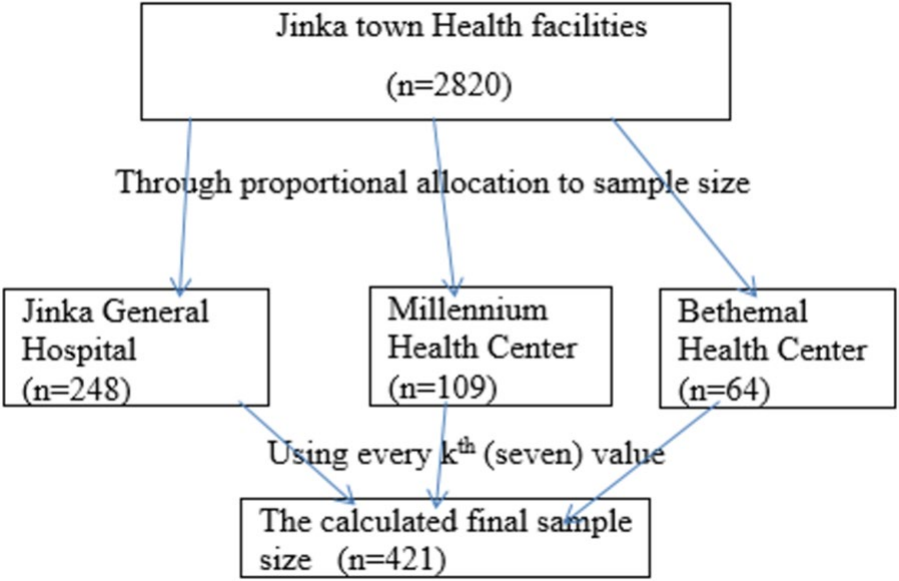
Figure showing the sampling procedure of the woman who came for FP service in pastoralist health facilities, Southern Ethiopia, 2022. Where n- number of women from each health facility

A 10% of non-response rate was also added and yielded a total sample size of 411. Then, the sample size was allocated proportionally to the number of each health facility within the town. A total of 2820 women visited FP service in Jinka town health facilities in the past 06 months (JGH 1660, Millennium Health center 734, and Bethemal Health center 426). Finally, systematic random sampling, using every kth value (every seven) was employed to select each woman from each health facility in Jinka town (Fig. 1).

### Data collection tools and procedure

Data were collected using structured, pretested, and interviewer-administered questionnaires. The questionnaires were initially adapted from the Ethiopian Demography and Health Survey (DHS) and the related published literature [8–16]. It addressed different factors such as sociodemographic factors (maternal age, place of residence, educational level, marital status, and occupational status), knowledge-related factors (Heard of BSE, where did you hear, common in your living area, detected early, the chance of survival, age screening recommended, family history of breast cancer), Attitudinal and practice-related factors.

### Data quality control

Before data collection, the questionnaires were translated into Arigna and Amharic and then retranslated back to English by the language-translation experts and health professionals to check for their consistency. The pretest was also done with 5% (n = 21) of the total sample size at the nearby health center to check the questionnaires for appropriateness, simplicity, clarity, understandability, and coherence before collecting the actual data. Moreover, Cronbach's alpha (0.86 and showing good internal consistency of items) were used to check for the reliability of the questions, and the validity of the content was also cross-checked by senior expert midwives working in a nearby hospital other than the study area. The training was given to the data collectors before the data collection process. Data collectors were midwives working in other health facilities nearby. The women were also informed about the benefits and risks associated with the study before data collection to give accurate results as possible.

Eight questions were used to assess the level of women's knowledge. Women who scored the mean and above were added and decisions were given. Women who answered four and above were considered good knowledge, and women who had below four were regarded as having poor knowledge. Likewise, seven questions were used to assess the attitude of women toward BSE using the Likert scale items, and the score of mean and above was used to decide the level of women's attitude toward BSE. Women who responded four and above were considered as having a good attitude, while below four were regarded as having a poor attitude toward BSE. Finally, eight questions were used to assess the level of women's BSE practice. The women who answered four and above considered as good BSE practice and had below four were taken as having poor women practice toward BSE. Moreover, Cronbach's alpha was assessed to check for the internal consistency of the items.

Our analysis included BSE practice as a dependent variable while considering sociodemographic, knowledge, attitudinal, and Practice related factors as independent variables.

### Data processing and analysis

The collected data was initially entered into Epi data version 7.2.2.6, then exported to SPSS version 25 for analysis. The descriptive statistics were computed such as means, frequencies, and proportions to summarize each relevant variable. Bivariate logistic regression analysis was carried out. Then, those variables that were significant at the bivariate level of analysis with *p* < 0.25 were taken into a multivariate logistic regression to identify the determinants. Finally, variables with a *p* value of < 0.05 in the final model were used to declare as determinants of BSE practices. Hosmer and Lemeshow test was also used to check for model fitness.

### Operational definitions

Breast self-examination (BSE)—the woman one's self-examination of the breasts to find any possible changes [5].

Breast self-examination Practice—ever performed BSE by the woman at least once a month [22].

Age 20 and American Cancer Society—the recommendations for women who want BSE by American cancer society for women aged 20 and above [5].

Knowledge of BSE: is the knowledge of the woman regarding what BSE means, how one can do it, and when one can do it using eight-question with the response of either 1 or 0 [8].

The attitude toward BSE—is what the women think and believes about BSE and was assessed using common Likert scales starting from strongly disagree (1) to strongly agree (5) [17].

The practice of breast self-examination: is the woman's act toward BSE and is assessed using eight potential questions with the response of either 1 or 0 for the right and wrong responses respectively [5, 15].

## Result

### Sociodemographic characteristics of the women

A complete interview data of 421 women who came for FP service was analyzed in pastoralist Health facilities, namely Jinka General Hospital, Millennium Health canter, and Bethemal Health center. Nearly half, 186 (44.2%) women were in the age range of 20–29 years with their mean (± SD) age of 25.43 ± 6.66 years. Regarding the women's place of residence, 238 (56.5%) were from urban.

Concerning the educational status of the women, 93 (22.1%) completed their primary level education. There were also 351 (83.4%) married and 52 (12.4%) single women. One hundred and seventy-five (41.6%) women were housewives. Forty-five (10.7%) women had a familial history of breast cancer (Table 1).

**Table 1:** Sociodemographic characteristics of the women who came for FP service in pastoralist Health facilities, Southern Ethiopia, 2022 (N = 421)

Variables	Categories	Frequency	Percentage
Maternal age	< 20	120	28.5
	20–29	186	44.2
	30–39	103	24.5
	> 39	12	2.9
Place of residence	Urban	195	46.3
	Rural	226	53.7
Maternal educational level	No formal education	219	52.0
	Primary education	93	22.1
	Secondary education	73	17.3
	Tertiary education	36	8.6
Maternal Marital status	Single	52	12.4
	Married	351	83.4
	Other	18	4.3
Occupational Status	Housewives	175	41.6
	Civil servant	108	27.3
	Self-employed	108	25.7
	Student	15	3.6
	Causal work	8	1.9
Family history of breast cancer	Yes	45	10.7
	No	376	89.3

*Others-Separated/widowed/divorced

### Knowledge of women toward BSE

Of the total women included in the analysis, almost half (49.9%) of the women heard of BSE previously. Among those women, 173 (53.8%) heard from health professionals. 356 (84.6%) women stated that breast cancer was not common in their living area. Three hundred and twenty-one (76.2%) knew that breast cancer is not detected early. Concerning the chance of survival from breast cancer, nearly one-fourth (24.0%) of the women knew that it is possible with BSE. Of the total women included in the study, 274 (65.1%) responded, screening for BSE should be started at an age of more than 20 years (Table 2).

**Table 2:** Knowledge of BSE among women who came for FP service in pastoralist Health facilities, Southern Ethiopia, 2022 (N = 421)

Variables	Categories	Frequency	Percentage
Heard of BSE	Yes	210	49.9
	No	211	50.1
Where did you hear about it	Health Professionals	106	50.5
	Peer group	10	4.8
	TV/Radio	71	33.8
	Schools including college/university	23	10.9
Breast cancer common in your living area	Yes	65	15.4
	No	356	84.6
Breast cancer detected early	Yes	100	23.8
	No	321	76.2
The chance of survival from breast cancer is possible with BSE	Yes	101	24.0
	No	320	76.0
Screening for BSE should be started at the age	20 years or less	147	34.9
	More than 20 years	274	65.1

### The attitude of the women toward BSE

**Fig. 2:**
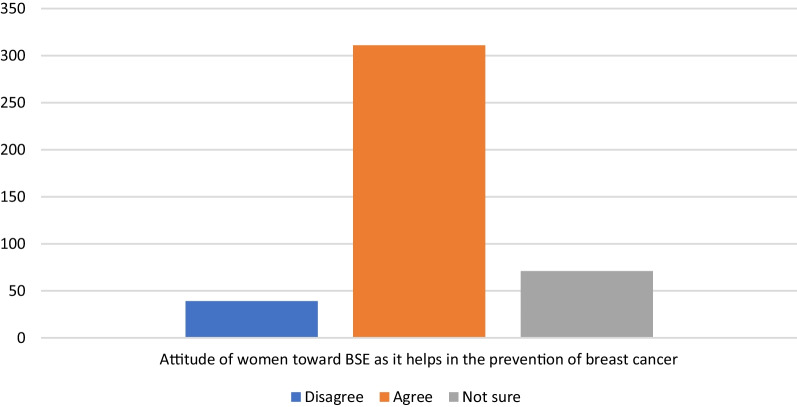
Figure showing the attitude of women toward BSE as it helps in the prevention of breast cancer among women who came for FP service in pastoralist health facilities, Southern Ethiopia, 2022

Two hundred and twenty-four (53.2%) women believed that BSE could be easily done. Of the total women, 293 (69.6) agreed that BSE was not a sexual activity initiation. Nearly three fourth (73.9%) of the women approved that BSE was important in the prevention of breast cancer. Furthermore, 179 (42.5%) women agreed as touching their breasts was obscene. More than half (59.4%) of the women want BSE because it did not lead to a positive cancer test. One hundred and forty-seven (34.9%) women believed that breast cancer was not hereditary. Two hundred and twenty-eight (54.2%) women reported that they were at risk of developing breast cancer (Fig. 2).

### Practices of breast self-examination

Eighty-nine (21.1%) women ever performed BSE in this study. One hundred and ninety-two (57.8%) women were not practiced BSE primarily because of thinking that they were healthy, followed by the fear of revealing breast cancer (35.2%). Regarding the way how BSE was performed, 76 (18.1%) women responded, palpating only with a single finger was possible. Among women who practiced BSE, nearly three fourth (73.0%) performed it in more than a quarter interval. Fifty-six percent (n = 50) of the women started practicing BSE by the age of 20–30 years. Of those women who performed BSE, 59 (66.3%) performed in the morning and nearly three-fourths performed it in their bathroom (Table 3).

**Table 3:** Practice of women toward BSE in pastoralist Health facilities, Southern Ethiopia, 2022 (N = 421)

Variables	Categories	Frequency	Percentage
Ever performed BSE	Yes	89	21.1
	Not	332	78.9
Reason for not performing BSE	Am healthy	192	57.8
	Afraid that it may reveal breast cancer	117	35.2
	Have not just decided	3	0.9
	It may be painful	11	3.3
	I feel shy	9	2.8
How often do you perform BSE in a year	More than once in a quarter	65	73.0
	Not very often	24	27.0
Age you practiced BSE	Less than 20	9	10.1
	20–30	50	56.1
	31–40	14	15.8
	> 41	16	18
How BSE performed	Palpating with one finger	76	18.1
	Palpate with palm and a minimum of three-finger	130	30.9
	Anyway can be possible	132	31.4
	No idea at all	83	19.7
Last time you performed BSE	Less than a week	1	1.1
	Less than three to six months	50	56.2
	More than a year ago	38	42.6
When do you perform BSE	Morning	59	66.3
	Afternoon	6	6.7
	Evening	11	12.4
	Anytime	13	14.6
Where do you perform BSE	In front of a mirror	22	24.7
	In the bathroom	67	75.3

### Determinants of breast self-examination practice

#### Bivariate and multivariable analysis for determinants of breast self-examination practice

In the bivariate analysis, four variables with a *p* value < 0.25 were selected as candidate variables for multivariable logistic regression analysis. These are; maternal place of residence, educational status, heard of BSE, and family history of breast cancer.

After controlling the effect of confounding variables in multivariable logistic regression analysis, women who resided in urban were associated with six times higher breast self-examination practice when compared with women who resided in rural (AOR = 6.79 CI = 3.40–13.56) *p* = 0.001. Women who had attained at least primary education and above have an eightfold increased chance of performing breast self-examination practice than their counterparts (AOR = 8.96 CI = 4.14–19.35) *p* = 0.001. The odds of women who heard about breast self-examination increased the likelihood of breast self-examination by four times more than their counterparts (AOR = 4.07 CI = 2.07–7.98) *p* = 0.001. When compared with families who had no previous history of breast cancer, having a family history of breast cancer increases the probability of breast self-examination by sevenfold (AOR = 7.46 CI = 3.27–17.00) *p* = 0.01 (Table 4).

**Table 4:** Determinants of breast self-examination practice among women who came for family planning service in pastoralist Health facilities, Southern Ethiopia, 2022

Variables	Category	Ever performed BSE		COR (95%CI)	AOR(95% CI)
		Yes	No		
Place of residence	Urban	75 (17.8)	120 (28.5)	9.46 (5.12–17.47)*	6.79 (3.40–13.56)**
	Rural	14 (3.3)	212 (50.4)	1	1
Educational status	No formal education	17 (4.0)	202 (48.0)	1	1
	Primary education	39 (9.3)	54 (12.8)	8.58 (4.50–16.33)*	8.96 (4.14–19.35)**
	Secondary Education	21 (5.0)	52 (12.4)	4.79 (2.36–9.74)*	2.27 (0.98–5.23)
	Tertiary Education	12 (2.9)	24 (5.7)	5.94 (2.53–13.92)*	1.93 (0.74–5.04)
Heard of BSE	Yes	70 (16.6)	140 (33.3)	5.05 (2.91–8.77)*	4.07 (2.07–7.98)**
	No	19 (4.5)	192 (45.6)	1	1
Family history of breast cancer	Yes	26 (6.2)	19 (4.5)	6.79 (3.54–13.03)*	7.46 (3.27–17.00)**
	No	63 (15.0)	313 (74.3)	1	

*Variables statistically significant at *p* value < 0.25

**Variables statistically significant at *p* value < 0.05

## Discussion

This study tried to examine the practice of breast self-examination among women who came for family planning services in pastoralist health facilities. In the current study, 21.1% (17.22–25.06) of the women had practiced breast self-examination. This shows that our current findings go in line with the studies from Modjo, and Gondar [8, 16]. However, the practice of BSE in the current study is lower than study conducted at Debre-Berhan University, West Gojjam, Nigeria, Eastern Uganda, Gamo Gofa, Debre tabor, west shoa, and Western Ethiopia [9, 11–14, 17–19]. This difference is mainly due to the difference in the study participants. In the case of the above four study areas, participants were students while the rest were health care professionals (HCPs) including health extension workers (HEWs). The students and workers population including HPs and HEW are very near to information either from their schools including university, or their working environment. The rate was also higher when compared with studies from Adwa, Kiewit, Kaffa, and Gojjam [15, 19, 23, 24]. This difference was due to differences in sociodemographic factors and healthcare-seeking behaviors. The study from Adwa indicated that 8.5% of the women were not completed their formal education, while the current study indicated more than half (52.0%) of the women were not. In addition to this, since the study area is one of the border areas and is inhabited mostly by a pastoralist community, healthcare-seeking behavior was poor when compared with other women from pastoralist communities [25].

In line with other studies, a woman who resided in an urban was over six times more likely to practice breast self-examination than women who resided in a rural. This statement is consistent with studies from Bulehora University and Systematic review and meta-analysis conducted in Africa [26, 27]. This is primarily because women who resided in an urban setting likely resulted with better awareness and good knowledge, which will bring the good practice of breast self-examination [20].

Similarly, women who had an educational status of primary education and above have an eight-fold increased chance of performing breast self-examination than their counterparts. This is consistent with studies conducted in Ghana and Modjo [16, 28]. The scientific justification for this could be higher educational attainment of the women resulted in a good woman attitude toward breast self-examination, which could bring the good practice of breast self-examination as well [6].

Women who heard about breast self-examination were associated with over four times more likely to practice breast self-examination than their counterparts. This statement is consistent with a study from Debretabor, Gamo Gofa, Modjo, Ghana, and West Arsi [10, 11, 13, 16, 28]. The possible rationale is that whenever women heard of breast self-examination, they will have the right information about its safety, easy procedure, and detection in its earlier stage before it goes to the advanced stage which will result in good practice of breast self-examination [5].

Finally, when compared with women who had no family history of breast cancer, a woman with a family history of breast cancer was associated with over sevenfold increased practice of breast self-examination. This statement is in line with findings from Debretabor, Gondar, and western Ethiopia [8, 11, 14]. The justification for this could be women who had a family history of breast cancer can get information easily from their family about the severity of breast cancer, risk factors, and preventive mechanisms, including the advantage of practicing breast self-examination [27].

### Limitations

Though, the study tried to identify key determinants of breast self-examination practice among women who came for family planning services in the study area. However, it has some limitations. Firstly, since the study was a cross-sectional study design, the causal association would not be predicted at all. Secondly, since the information was collected when the woman was not performing BSE, the introduction of recall and social desirability bias would be inevitable. Thirdly, because the study was conducted in pastoralist health facilities, it does not necessarily reflect another context. Despite all these limitations, our study identified key determinants of BSE practices in the study area. The study findings can be addressed if carefully planned and implemented at all levels of health care.

## Conclusion

Our study showed that women's practice of BSE was lower when compared with the local studies. Women who heard of BSE resided in urban, attained at least primary education and above, and had a family history of breast cancer were significantly associated with women's practice of BSE. So, we recommend health care professionals and others working in the area increase awareness about breast cancer, including its risk, and the need for BSE.

## Data Availability

The dataset used and/or analyzed during the current study are available from the corresponding author on reasonable request.

## Abbreviations

ACS: American Cancer Society
AOR: Adjusted Odds Ratio
BHC: Bethemal Health Center
BSE: Breast Self-Examination
CBE: Clinical breast Examination
EDHS: Ethiopian Demographic and Health Survey
FMOH: Federal Ministry of Health
FP: Family Planning
HEW: Health Extension workers
HP: Health Professionals
IRB: Institutional Review Board
JGH: Jinka General Hospital
MHC: Millennium Health Center
NCD: Non-communicable Disease
SDG: Sustainable Development Goals
SPSS: Statistical Software for Social Science
SSA: Sub-Saharan Africa
SoZHD: South Omo Zone Health Department
